# Supplementary material for: Engineering of Ni(OH)2 Modified Two-Dimensional ZnIn2S4 Heterostructure for Boosting Hydrogen Evolution under Visible Light Illumination
Source: Nanomaterials (Basel). 2022 Mar 13;12(6):946. doi: 10.3390/nano12060946 (PMC8949192; doi:10.3390/nano12060946)
Supplement: Supplementary file 1 [file nanomaterials-12-00946-s001.zip › nanomaterials-1538519-supplementary.pdf]

nanomaterials

Supplementary materials

Engineering of $Ni(OH)_2$ Modified Two-Dimensional $ZnIn_2S_4$ Heterostructure for Boosting Hydrogen Evolution under Visible Light Illumination

Huan Wang [1,2,*], Baorui Shao [2], Yaodan Chi [1], Sa Lv [1], Chao Wang [1], Bo Li [2], Haibin Li [2], Yingui Li [2] and Xiaotian Yang [1,3,*]

[1] Key Laboratory for Comprehensive Energy Saving of Cold Regions Architecture of Ministry of Education, Jilin Jianzhu University, Changchun 130118, China; chiyaodan@jlju.edu.cn (Y.C.); lvsa82@163.com (S.L.); wangchao@jlju.edu.cn (C.W.)

[2] Department of Materials Science, Jilin Jianzhu University, Changchun 130118, China; br13180828503@163.com (B.S.); q11833395833@163.com (B.L.); LHB980215@163.com (H.L.); l15584271740@163.com(Y.L.)

[3] Department of Chemistry, Jilin Normal University, Siping 136000, China

* Correspondence: wanghuan@jlju.edu.cn (H.W.); hanyxt@163.com (X.Y.); Tel.: +86-0431-84566327 (H.W.); +86-0434-3295002 (X.Y.)

Table S1. The actual chemical compositions of the prepared samples.

	Zn (ppm)	In (ppm)	S (ppm)	Ni (ppm)	$Ni(OH)_2$ wt% in the composite
$ZnIn_2S_4$	156.700	559.400	242.500	NA	
2.7 mg$Ni(OH)_2/ZnIn_2S_4$	851.200	2799.000	1888.000	3.589	0.10
6.1 mg$Ni(OH)_2/ZnIn_2S_4$	691.100	2705.000	1626.000	6.141	0.22
10 mg$Ni(OH)_2/ZnIn_2S_4$	760.900	2532.000	1619.000	11.56	0.37
20 mg$Ni(OH)_2/ZnIn_2S_4$	732.300	2664.000	1639.000	22.180	0.74

Table S2. BET surface area and pore size distribution of different samples.

	BET Surface Area (m^2/g)	BJH Adsorption average pore width (nm)	BJH Desorption average pore width (nm)
$ZnIn_2S_4$	0.5099	140.6599	31.8727
0.1 wt% $Ni(OH)_2/ZnIn_2S_4$	3.572	15.6966	38.223
0.22 wt% $Ni(OH)_2/ZnIn_2S_4$	8.505	65.8517	21.3987
0.37 wt% $Ni(OH)_2/ZnIn_2S_4$	101.33	21.3987	5.9312
0.74 wt% $Ni(OH)_2/ZnIn_2S_4$	124.65	20.0394	10.1336
$Ni(OH)_2$	24.44	69.1154	51.8633

Table S3. Photocatalytic H_2 evolution results of some related works reported recently.

	Cocatalys	Electron donor	Solvent	Reaction temperature	Rate	Incident light	Ref.
$Ni(OH)_2$/ ZnIn2S4	$Ni(OH)_2$	lactic acid	H_2O	Ambient temperature	4430 μmol h^{-1} g^{-1}	λ> 420 nm (300 W Xe lamp with an ultraviolet cutoff filter)	RSC Adv., 2021, 11, 12442-12448
$Ni(OH)_2$/ ZnIn2S4	$Ni(OH)_2$	triethanolamine	H_2O	Ambient temperature	401 μmol h^{-1} g^{-1}	λ> 420 nm (400 W Xe lamp with an ultraviolet cutoff filter)	Sustainable Energy Fuels, 2020, 4, 750-759
$ReS_2/ZnIn_2S_4$	ReS_2	triethanolamine	H_2O	Ambient temperature	1859 μmol h^{-1} g^{-1}	λ> 420 nm (300 W Xe lamp with an ultraviolet cutoff filter)	Journal of Alloys and Compounds 2021, 873, 159850
$ZnIn_2S_4$@ $CuInS_2$	$CuInS_2$	Na_2S/Na_2SO_3	H_2O	Ambient temperature	1168μmol $h^{-1}g^{-1}$	λ> 420 nm (300 W Xe lamp with an ultraviolet cutoff filter)	J. Phys. Chem. C2020, 124, 5934-5943
CoNi - $ZnIn_2S_4$	NA	ascorbic acid	H_2O	0℃	3336.6 μmol ·$h^{-1}g^{-1}$	λ> 420 nm(300 W Xe lamp with an ultraviolet cutoff filter)	ACS Sustainable Chem. Eng. 2019, 7, 20190-20201
$CuS/CdIn_2S_4/ZnIn_2S_4$	Pt	Na_2S/Na_2SO_3	H_2O	Ambient temperature	233.9μmol $h^{-1}g^{-1}$	λ> 420 nm (300 W Xe lamp with an ultraviolet cutoff filter)	ACS Sustainable Chem. Eng.2016, 4, 6680-6688
$Ni(OH)_2$/ $ZnIn_2S_4$	$Ni(OH)_2$	triethanolamine	H_2O	Ambient temperature	4640μmol $h^{-1}g^{-1}$	λ> 420 nm (300 W Xe lamp with an ultraviolet cutoff filter)	This work

Table S4. Detailed time-resolved transient PL decay fitting data of $ZnIn_2S_4$ and 0.37wt% $Ni(OH)_2/ZnIn_2S_4$, respectively.

	x^2	f_1 (%)	$\tau_1(ns)$	f_2 (%)	$\tau_1(ns)$	f_3 (%)	$\tau_1(ns)$	Average τ (ns)
$ZnIn_2S_4$	1.1380	38.41	0.3962	46.48	2.0923	15.11	3.9230	1.68
0.37wt% $Ni(OH)_2/ZnIn_2S_4$	1.0732	39.41	0.3345	46.06	1.4037	14.53	4.4384	1.42

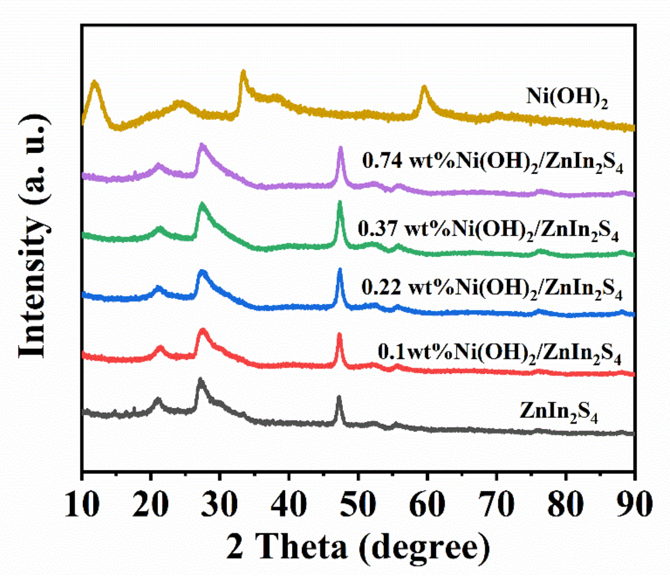

Figure S1. XRD patterns of the samples after the preheating treatment before BET measurements.

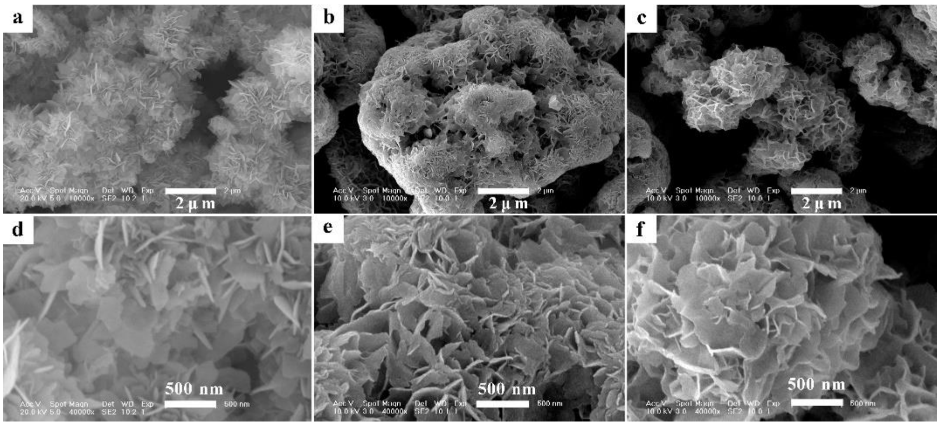

Figure S2. SEM image and enlarged SEM images of pure $Ni(OH)_2$ sheets (**a, d**), $ZnIn_2S_4$ (**b, e**) and 0.37 wt% $Ni(OH)_2/ZnIn_2S_4$ composite (**c, f**).

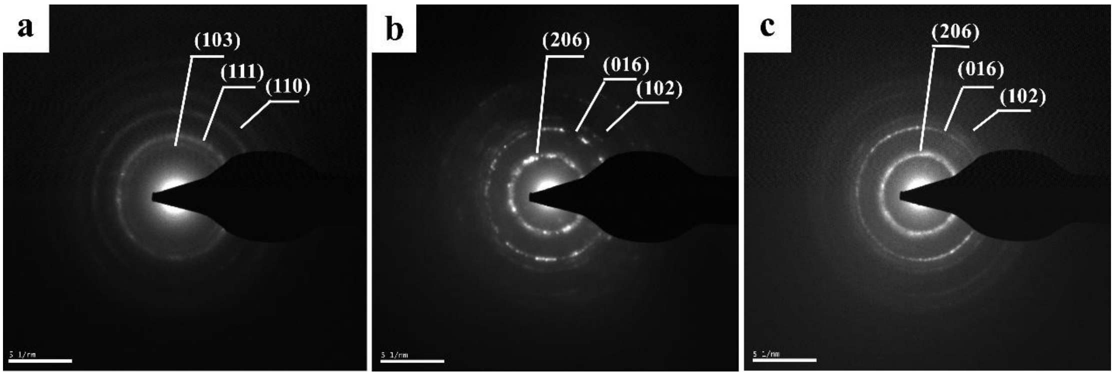

Figure S3. SEAD images of $Ni(OH)_2$ (**a**), $ZnIn_2S_4$ (**b**), and 0.37 wt% $Ni(OH)_2/ZnIn_2S_4$(**c**), respectively.

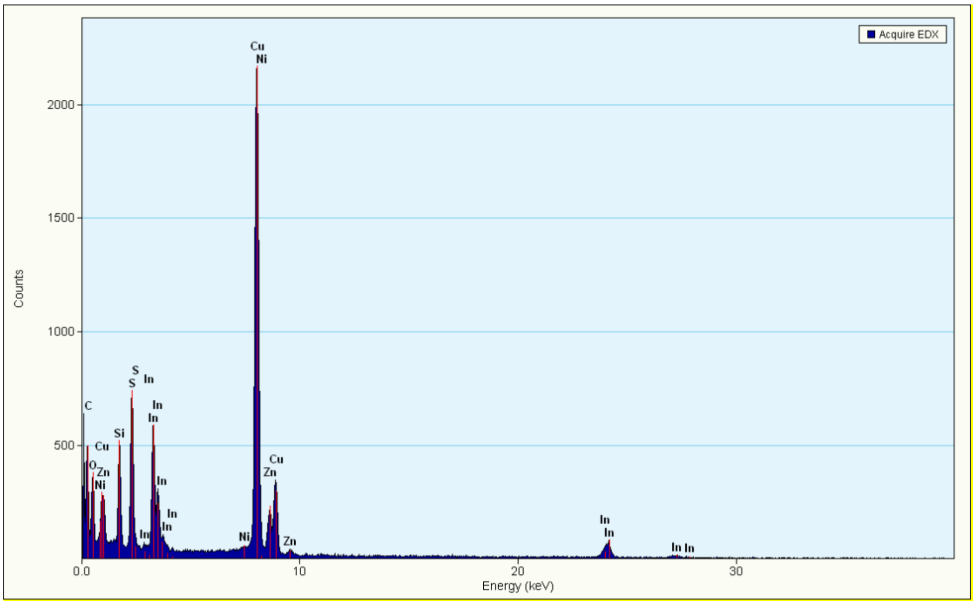

Figure S4. EDX spectrum of the 0.37 wt% $Ni(OH)_2/ZnIn_2S_4$ composite.

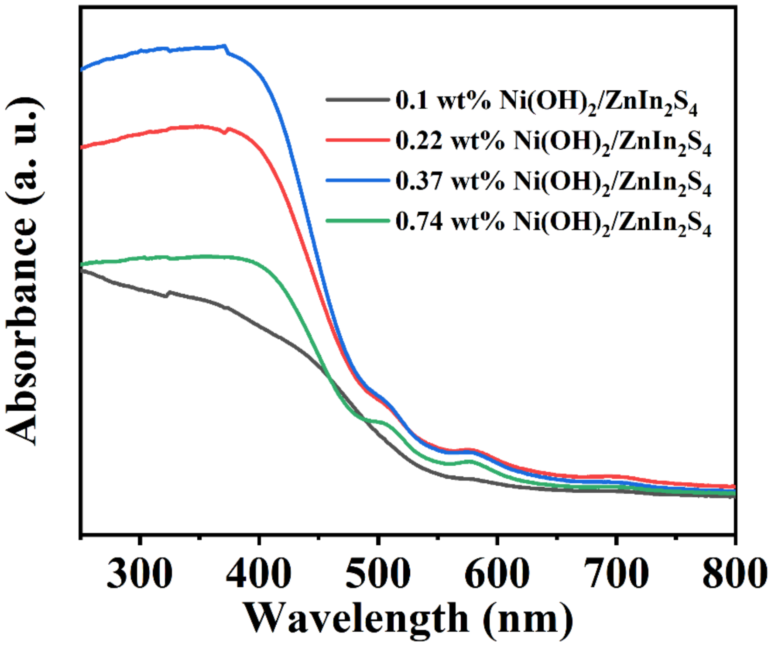

Figure S5. UV-Vis diffuse reflectance spectra of $Ni(OH)_2/ZnIn_2S_4$ composite samples with different amounts of $Ni(OH)_2$ introduced.

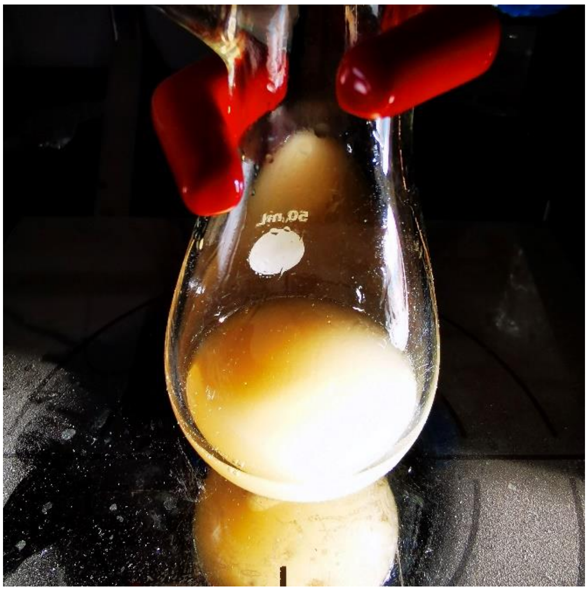

Figure S6. Photograph of the photocatalytic reactor with 0.37 wt% $Ni(OH)_2/ZnIn_2S_4$ as photocatalyst during the reaction.
